# Врожденная дисфункция коры надпочечников, обусловленная дефицитом 11β-гидроксилазы: поздняя диагностика и смена пола у ребенка двух лет

**DOI:** 10.14341/probl12749

**Published:** 2021-09-26

**Authors:** Н. Ю. Райгородская, Е. П. Новикова, А. Н. Тюльпаков, М. A. Карева, Н. А. Николаева, Н. В. Болотова

**Affiliations:** Саратовский государственный медицинский университет им. В.И. Разумовского; Саратовский государственный медицинский университет им. В.И. Разумовского; Национальный медицинский исследовательский центр эндокринологии; Медико-генетический научный центр им. академика Н.П. Бочкова; Национальный медицинский исследовательский центр эндокринологии; Саратовский государственный медицинский университет им. В.И. Разумовского; Саратовский государственный медицинский университет им. В.И. Разумовского

**Keywords:** врожденная дисфункция коры надпочечников, дефицит 11β-гидроксилазы, мутации гена CYP11B1, преждевременное половое развитие

## Abstract

Дефицит 11β-гидроксилазы — редкое заболевание с аутосомно-рецессивным типом наследования, обусловленное нарушением стероидогенеза в надпочечниках в результате патогенных мутаций в гене CYP11B1. Основные клинические проявления определяются дефицитом кортизола, гиперпродукцией адренокортикотропного гормона, избыточной секрецией андрогенов и накоплением 11-деоксикортикостерона, что приводит к развитию артериальной гипертензии. В процессе диагностического поиска важно учитывать этническую принадлежность пациента, так как частота заболевания и распространенность мутаций имеют особенности среди отдельных этнических групп. В статье представлен клинический случай дефицита 11β-гидроксилазы в результате компаунд-гетерозиготных мутаций в гене CYP11B1 у пациентки тюркского происхождения. На примере данного наблюдения можно проследить клинические проявления и развитие осложнений классической формы дефицита 11β-гидроксилазы, этапы дифференциальной диагностики пациентов с дефицитом 21-гидроксилазы.

## Abstract (en)

11β-hydroxylase deficiency is a rare autosomal recessive disorder due to impaired steroidogenesis in the adrenal cortex caused by pathogenic mutations in the CYP11B1 gene. The main clinical manifestations are determined by a deficiency of cortisol, ACTH hyperproduction, excessive androgens secretion and the accumulation of 11-deoxycorticosterone, which leads to the development of arterial hypertension. In the diagnostic search, it is important to take into account the ethnicity of the patient, since the frequency of the disease and the prevalence of mutations differ between ethnic groups. The article presents a clinical case of 11β-hydroxylase deficiency as the result of compound heterozygous mutations in the CYP11B1 gene in a patient of Turkic origin. This case shows the clinical manifestations and the development of complications of 11β-hydroxylase deficiency, the stages of differential diagnosis of patients with 21-hydroxylase deficiency.

## АКТУАЛЬНОСТЬ

Дефицит 11β-гидроксилазы — один из вариантов нарушения стероидогенеза в надпочечниках, обусловленный патогенными мутациями в гене CYP11B1, кодирующем функцию данного фермента. В результате дефицита 11β-гидроксилазы нарушается превращение 11-деоксикортизола в кортизол и 11-деоксикортикостерона (ДОК) в кортикостерон и альдостерон [1]. Это обуславливает дефицит кортизола и гиперпродукцию адренокортикотропного гормона (АКТГ). Гиперстимуляция надпочечников, в свою очередь, приводит к избыточной секреции андрогенов, накоплению 11-­деоксикортизола и 11-деоксикортикостерона (11-ДОК), который является минералокортикоидом. Гиперпродукция 11-ДОК вызывает задержку натрия в организме и является частой причиной артериальной гипертензии. Дефицит 11β-гидроксилазы называют гипертонической формой врожденной дисфункции коры надпочечников (ВДКН). В постнатальном периоде артериальная гипертензия и гипернатриемия, как правило, не определяются из-за транзиторной резистентности новорожденных и детей раннего возраста к минералокортикоидам [2][3]. Может определяться ­повышенный уровень 17-ОН-прогестерона, предположительно, как результат подавления 21-гидроксилазы при высоком уровне 11-деоксикортизола [4]. Особенности детей раннего возраста обусловливают трудности дифференциальной диагностики 11β-гидроксилазы и дефицита 21-гидроксилазы. Представленное нами наблюдение отражает клиническую ситуацию поздней диагностики гипертонической формы ВДКН и этапы ­диагностического поиска.

## ОПИСАНИЕ СЛУЧАЯ

Родители ребенка впервые обратились за медицинской помощью в возрасте 2 лет 4 мес с жалобами на отсутствие яичек в мошонке, раннее оволосение лобковой области, угревую сыпь. Из анамнеза стало известно, что ребенок родился от неродственного брака родителей-­азербайджанцев, от второй беременности, протекавшей физиологически. Первая беременность закончилась рождением здорового мальчика.

Ребенок родился доношенным с ростом 50 см и массой 3050 г, пол при рождении определен как мужской, обращало на себя внимание только отсутствие яичек в мошонке. В возрасте 1 года мама заметила наличие единичных светлых волос на лобке и увеличение полового члена, появление угревой сыпи на спине, груди и верхних конечностях; с 1,5 года — бурный рост и потемнение волос на лобке, гиперпигментацию мошонки. В возрасте 2 лет ребенок был осмотрен урологом по причине отсутствия яичек в мошонке и направлен на обследование в детское эндокринологическое отделение.

При первичном осмотре в возрасте 2 лет 4 мес обнаружены ускоренные темпы роста, рост составлял 96 см, SDS роста = +2,35 при гармоничном физическом ­развитии: масса — 15 кг, ИМТ — 16,3 кг/м2, SDS ИМТ= -0,3; гиперпигментация кожи, угревая сыпь на лице, груди, верхних конечностях; хорошее развитие мышц верхнего плечевого пояса, конечностей. Обращали на себя внимание особенности поведения ребенка: беспокойство, раздражительность, агрессивность, односложные ответы на вопросы. Низкий тембр голоса. Артериальное давление 85/50 мм рт.ст., что соответствовало 50-му перцентилю относительно показателей физического развития.

Наружные половые органы имели маскулинное строение и соответствовали 5-й степени вирилизации по Прадеру, пигментированы. Гонады при пальпации не определялись. Развитие лобкового оволосения соответствовало стадии Р3 по классификации Таннера.

Обследование ребенка было начато с кариотипирования и определения костного возраста. Установлен кариотип 46,XX. Костный возраст соответствовал 9 годам, то есть опережал паспортный на 6,5 года. При ультразвуковом исследовании малого таза была визуализирована матка 14×9,7×10 мм с шейкой 15,5 мм и влагалище, расширенное за счет анэхогенного содержимого. Слева от матки визуализировано образование объемом 0,66 см3, неоднородное по структуре за счет анэхогенных включений и локальных участков фиброза, которое было расценено как яичник. При УЗИ надпочечников объемных образований не обнаружено.

Вирильный синдром у девочки с рождения, выраженное опережение костного возраста ­позволили предположить наличие ВДКН, вероятнее всего, обусловленное дефицитом 21-гидроксилазы. Для подтверждения диагноза было проведено гормональное обследование, определены: повышенный уровень 17-ОН-прогестерона — 45,1 нмоль/л (0,27–6,1); высокие показатели тестостерона — 10 нмоль/л (0,1–1,0) и АКТГ — 705 пг/мл (4,0–46,0). Показатели электролитов в сыворотке крови соответствовали нормальным значениям: калий — 4,47 ммоль/л, ­натрий — 139,4 ммоль/л. Уровень прямого ренина — 1,32 мкМЕ/мл (2,8–39,9).

На основании проведенного обследования установлен диагноз: «Врожденная дисфункция коры надпочечников, вирильная форма». Родителям предоставлена информация об имеющемся заболевании и прогнозе развития ребенка, о необходимости проведения заместительной гормональной терапии и целесообразности воспитания ребенка в женском гражданском поле. Начата заместительная терапия глюкокортикоидами (гидрокортизон) в суточной дозе 15 мг/м2, разделенной на 3 приема в течение суток. В возрасте 2 лет 10 мес проведена документальная смена гражданского пола, ребенок зарегистрирован в женском поле; в 2 года 11 мес в клинике детской хирургии Саратовского ГМУ проведен 1-й этап феминизирующей пластики наружных половых органов.

Повышенный уровень 17-ОН-прогестерона в сочетании с клинической картиной заболевания явились основанием для молекулярно-генетической диагностики на частые мутации гена CYP21A2 c целью подтверждения дефицита 21-гидроксилазы — наиболее частого варианта вирильного синдрома у девочек. Однако при проведении аллель-специфической полимеразной цепной реакции (ПЦР) на 12 точечных мутаций в гене CYP21A2 частые мутации не обнаружены.

Продолжалось наблюдение за ребенком на фоне проведения заместительной терапии. В результате лечения глюкокортикоидами снизились показатели 17-ОН-прогестерона — 25,1 нмоль/л (0,27–6,1) и ­тестостерона — 2,35 нмоль/л (0,1–1,0). Однако клинически не удавалось достичь хорошей компенсации: сохранялись гиперпигментация, гирсутизм, лобковое оволосение, ускоренные темпы роста — 8 см/год, прогрессирование костного возраста. Учитывая клиническую картину ВДКН с выраженной андрогенизацией при умеренном повышении 17-ОН-прогестерона, отсутствие минералокортикоидной недостаточности и низкие показатели ренина плазмы, а также отсутствие частых мутаций в гене CYP21A2, было принято решение о необходимости диагностики дефицита 11β-гидроксилазы. При секвенировании гена CYP11B1 выявлены 2 гетерозиготные мутации: c.896T>C p.L299P и c.370delG p.H125TfsX8. Установлен дефицит 11β-­гидроксилазы, обуславливающий развитие гипертонической формы ВДКН.

С 5,5 года появилась тенденция к повышению артериального давления: 110/60–120/80 мм рт.ст., что соответствовало 90–95 перцентилю относительно показателей физического развития. К этому времени девочка имела рост 116 см, SDS +1,3, массу тела 19 кг, ИМТ 14 кг/м2. Костный возраст соответствовал 11 годам. Доза гидрокортизона на момент осмотра составила 13 мг/м2 и была увеличена до 15,6 мг/м2/сут.

В 7,5 года госпитализирована с жалобами на увеличение молочных желез, которое мать отмечала на протяжении последнего года. Появление железистой ткани в области молочных желез отмечалось на фоне сохраняющихся проявлений гиперандрогении и прогрессирования костного возраста. Рост 132 см, SDS роста +1,83, скорость роста 8,2 см/год, костный возраст соответствовал 12 годам, формула полового развития — Ma2P3. Выявлено прогрессирование артериальной гипертензии — 125/80–130/80 мм рт.ст. Проявления декомпенсации были связаны с нарушением режима приема гидрокортизона в течение последнего месяца. При гормональном обследовании: тестостерон — 6,1 нмоль/л, 17-ОН-прогестерон — 36,5 нмоль/л, лютеинизирующий гормон (ЛГ) — 0,36 мЕд/мл, фолликулостимулирующий гормон (ФСГ) — 1,13 мЕд/мл. Учитывая клинические проявления преждевременного полового развития при значительном прогрессировании костного возраста, была проведена проба с аналогом гонадотропин-рилизинг-гормона. Максимальный выброс ЛГ через 4 ч после введения ГнРГ составил 22 мЕд/мл, ФСГ — 17,8 мЕд/мл. Результаты пробы соответствовали гонадотропинзависимому преждевременному половому развитию.

Проведена коррекция глюкокортикоидной терапии, назначен гидрокортизон из расчета 20 мг/м2, отрегулирован режим приема препарата. На фоне проводимого лечения уменьшилась угревая сыпь, нормализовалось АД, снизился уровень тестостерона до 1,9 нмоль/л. С целью купирования симптомов преждевременного полового развития, предупреждения прогрессирования костного возраста начато лечение диферелином 3,75 мг 1 раз в 28 дней.

## ОБСУЖДЕНИЕ

ВДКН, обусловленная дефицитом 11β-гидроксилазы, относится к орфанным заболеваниям — 1:100 000–1:200 000 новорожденных и составляет около 5% ­среди всех вариантов ВДКН в европейской популяции [4]. Исключение составляют отдельные этнические группы. Наибольшую распространенность дефицита 11β-­гидроксилазы имеет популяция марокканских евреев, живущих в Израиле, 1:5000–1:7000 новорожденных [5]. Высокая частота заболевания выявлена среди народов тюркского происхождения (13,5% среди всех форм ВДКН), к которым относятся и азербайджанцы [6].

Заболевание наследуется по аутосомно-рецессивному типу. Для фенотипической реализации дефицита 11β-гидроксилазы необходимо наличие гомозиготной или 2 компаунд-гетерозиготных мутаций. Мутация с.896T>C L299P, обнаруженная у нашего пациента, является наиболее частой мутацией в гене CYP11B1 в турецкой популяции [7].

Представленная пациентка имела классические клинические проявления дефицита 11β-гидроксилазы: сочетание гиперандрогении и артериальной гипертензии, которая манифестировала в возрасте 5,5 года. На этапе первичного обследования у девочки отмечались нормальные показатели артериального давления и клинико-гормональные проявления заболевания, сходные с дефицитом 21-гидроксилазы: вирильный синдром, опережение костного возраста, высокий уровень тестостерона и повышенный уровень 17-ОН-прогестерона в сыворотке крови. При проведении молекулярно-генетического обследования методом аллель-специфической ПЦР частые мутации в гене CYP21A2 не были обнаружены. Для исключения дефицита 21-гидроксилазы требовалось проведение полного секвенирования гена CYP21A2. Однако низкие значения ренина плазмы при высоком уровне тестостерона и АКТГ позволили заподозрить гипертоническую форму ВДКН, обусловленную дефицитом 11β-гидроксилазы [10]. Гормональным маркером данного состояния является определение 11-дезоксикортикостерона в сыворотке крови [8–10], что не было сделано по причине трудной доступности данного лабораторного исследования. Таким образом, низкий уровень ренина плазмы при высоком уровне АКТГ и отсутствие частых мутаций в гене CYP21A2 определили показания для молекулярно-генетического обследования на дефицит 11β-гидроксилазы. При секвенировании гена CYP11B1 были выявлены 2 гетерозиготные мутации: миссенс-мутация — c.896T>C p.L299P и делеция нуклеотида, приводящая к сдвигу рамки считывания и возникновению стоп-кодона -c.370delG p.H125TfsX8. Установлен дефицит 11β-гидроксилазы. Отсутствие артериальной гипертензии у ребенка раннего возраста можно объяснить транзиторной резистентностью к минералокортикоидам [2].

Миссенс-мутация c.896T>C p.L299P впервые была описана в 2005 г. у мальчика 2,5 года, уроженца ­Ирака, не получавшего лечение и имеющего классические проявления вирильной формы ВДКН, высокий АКТГ и умеренно повышенный уровень 17-ОН-прогестерона — 8,5 нмоль/л [11]. При изучении авторами L299P мутации in vitro было показано снижение ферментативной активности 11β-гидроксилазы до 1,2±0,9%. Делеции CYP11B1 также приводили к тяжелой потере активности 11β-гидроксилазы. По данным Международного Консорциума редких стероидных заболеваний, представившего результаты генетического обследования 108 пациентов с дефицитом 11β-гидроксилазы и анализ генотип-фенотипических корреляций, мутация L299P приводит к выраженному снижению стабильности фермента и является предиктором классической формы заболевания с тяжелыми клиническими проявлениями [9].

В нашем случае первичная диагностика была проведена ребенку в возрасте 2,5 года. Позднее начало терапии и недостаточная комплаентность родителей привели к быстрому опережению костного возраста, раннему появлению вторичных половых признаков. Для купирования проявлений истинного преждевременного полового развития в возрасте 7 лет потребовалось назначение аналога гонадотропин-рилизинг-гормона. Проявлениями декомпенсации и недостаточной эффективности лечения также явились ранняя манифестация и прогрессирование артериальной гипертензии.

## ЗАКЛЮЧЕНИЕ

Таким образом, наличие у нашего пациента компаунд-гетерозиготных мутаций (миссенс-мутации L299P и мутации со сдвигом рамки считывания) в гене CYP11B1 соответствует классической клинической картине с полными клиническими проявлениями. Поздняя постановка диагноза и несвоевременное начало терапии привели к раннему развитию артериальной гипертензии и осложнений в виде выраженного опережения костного возраста, истинного преждевременного полового развития.

## ДОПОЛНИТЕЛЬНАЯ ИНФОРМАЦИЯ

Источники финансирования. Молекулярно-генетические исследования были проведены в лаборатории отделения наследственных эндокринопатий ФГБУ «НМИЦ эндокринологии» при финансовой поддержке Фонда поддержки и развития филантропии «КАФ» в рамках Национальной благотворительной программы помощи детям с эндокринными заболеваниями «Альфа-Эндо».

Конфликт интересов. Авторы декларируют отсутствие явных и потенциальных конфликтов интересов, связанных с содержанием настоящей статьи.

Участие авторов. Райгородская Н.Ю. — сбор и анализ материала, написание текста статьи; Новикова Е.П.— анализ материала, описание клинического случая; Болотова Н.В. — анализ материала, создание концепции изложения, редактирование текста статьи; Карева М.А.  — анализ материала, анализ данных литературы, подготовка раздела «Обсуждение»; Тюльпаков А.Н. — анализ материала, выполнение генетических исследований, формирование генетического заключения, редактирование текста статьи; Николаева Н.А. — сбор и систематизация клинического материала, сбор и анализ литературы. Все авторы внесли значимый вклад в проведение исследования и подготовку статьи, прочли и одобрили финальную версию статьи перед публикацией. Все авторы одобрили финальную версию статьи перед публикацией, выразили согласие нести ответственность за все аспекты работы, подразумевающую надлежащее изучение и решение вопросов, связанных с точностью или добросовестностью любой части работы.

Согласие пациента. Мать ребенка добровольно подписала информированное согласие на публикацию персональной медицинской информации в обезличенной форме в журнале «Проблемы эндокринологии».
